# Distribution of the Highest Plantar Pressure Regions in Patients with Diabetes and Its Association with Peripheral Neuropathy, Gender, Age, and BMI: One Centre Study

**DOI:** 10.1155/2019/7395769

**Published:** 2019-07-09

**Authors:** Edyta Sutkowska, Krzysztof Sutkowski, Michał Sokołowski, Edward Franek, Szymon Dragan

**Affiliations:** ^1^Department and Division of Medical Rehabilitation, Wroclaw Medical University, Poland; ^2^Department of General, Minimally Invasive and Endocrine Surgery, Wroclaw Medical University, Poland; ^3^Mossakowski Clinical Research Centre, Polish Academy of Sciences, Warsaw, Poland; ^4^Department and Clinic of Orthopaedic and Traumatologic Surgery, Wroclaw Medical University, Poland

## Abstract

The abnormal plantar pressure distribution and value play a key role in the formation of plantar calluses and diabetic foot ulcer. The prevalence of the highest pressure different distribution and its association with various factors among patients with diabetes is not well known. The study purpose was to evaluate the prevalence of different regions for the highest pressure on the sole and its association with selected factors among patients with diabetes. Medical records of nonulcer patients were retrospectively analysed. The relationship between pressure patterns on the sole obtained during a pedobarographic test as a semiquantitative assessment with colourful print analysis and neuropathy, gender, age, and BMI was searched. The most common location of the highest pressure was the central part of the forefoot. No association was found between the different highest pressure regions and age, sensory neuropathy, calluses, and foot deformities. The highest pressure on the lateral part of the foot and midfoot was observed more often in females and in patients with a BMI ≥ 35. The prevalence of the highest pressure on the forefoot was more common in patients with a BMI < 35. *Conclusions*. The most frequent regions of the highest pressure on the sole in patients with diabetes were the central part of the forefoot (2-3 metatarsal heads) with no simple relationship to the assessed variables other than BMI < 35. Female gender and higher BMI seem to be responsible for shifting the place of the highest pressure to other places of the foot.

## 1. Introduction

The foot is the most inferior-located part of the body that bears weight. The arrangement of bones, muscles, and joints allows mobility by absorbing and supporting vigorous pressure during standing and walking. Despite the initial overthrow of the “tripod” theory of load distribution (three points where the foot contact the ground) in 1987 by Cavanagh et al. [1], based on the description of human anatomy as well as on work presented by Taha et al. in 2016 [2], it seems that physiologically, there are three main points of the highest load on the sole: central part of the heel and the 1st and 4th-5th metatarsal heads. The physiological pressure distribution pattern on the sole is nearly symmetric and gives the foot, and thus our body, optimal stabilization.

Pressure distribution and value beneath the plantar surface depends on, e.g., body weight [3], age [4], and foot abnormalities secondary to disease [5, 6]. In population with diabetes, also poor control of the disease seems to be responsible for unnormal foot pressure [7, 8]. It is a known fact that the increased pressure in patients with diabetes peripheral neuropathy may be responsible for foot ulceration [9–19] and that foot ulcers occur mainly under the metatarsal heads [9, 20, 21]. In one of the recent big meta-analysis [22] which was dedicated to the relationship between ulceration and neuropathy, the authors stressed that people with diabetes peripheral neuropathy and previous ulceration demonstrate higher plantar pressure compared to those with diabetes peripheral neuropathy but no ulceration history, which seems to be understandable. However, in this meta-analysis, the authors also demonstrated results which showed that patients with diabetes and active foot ulceration do not demonstrate an elevation in plantar pressures compared to those with neuropathy and no ulceration history. These surprising results of meta-analysis confirm how complicated are the relationships between the various studied dependencies on the foot. The most likely explanation of such an observation which the authors' proposed is the offloading theory which suggest that people with active ulceration protect the part of the foot where the ulceration exist which has an impact on the pressure measurement results. It is assumed currently that, appeared to foot ulcers, high plantar pressure must coexist with neuropathy [23–25]. The other author [26], however, observed 28% incidence of ulceration in patients with peripheral neuropathy and high plantar pressure but did not confirm the presence of ulcers in patients with neuropathy but without abnormal plantar pressure. This suggests that abnormal value of foot pressure as well as neuropathy could play an important role in the formation of plantar ulcers independently.

Pathological foot structure and abnormalities during the gait are responsible for incorrect pressure on the sole, and it should be emphasized that both are responsible for the risk of diabetic foot [27]. The result of the first is a disorder of standing and walking mechanics, which can have an important impact on inappropriate foot peak plantar pressure value and location [28]. The plantar pressure pattern and value can be determined by a pedobarographic examination [29]. The pedobarograph is a device which converts the applied pressure into a visible light pattern with a pressure measurement. The important role of pedobarography, as a diagnostic procedure, in plantar pressure pattern and value creating during stance and/or gait is confirmed in many studies. Unfortunately, as was mentioned by Fernando et al. [30], there is no standardized protocol for this assessment up to now. In summary, because of the complexity of the problem and due to the lack of good quality studies, there is no clear information on how to assess the plantar pressure in the guidelines which is dedicated to the diabetic foot prevention and treatment [31]. Studies most often emphasize the role of the dynamic test (during walking) which requires special insoles with pressure sensors or floor-based foot pressure measurement devices and the computer software to interpret the results. The subjects need special training for the optimal measurements before the dynamic pressure assessment, which is time consuming. The evaluation also requires additional skills [30]. For this reason, most dynamic pressure measurement devices are not routinely used as screening tools in daily clinical practice in patients with diabetes and are rather used for clinical research. Additionally, despite of this clinical research, the research problems with study interpretation could also be found, e.g., as in one study [32], 12 steps per foot were required; the other authors in previous studies analysed less number of steps or gave no detailed information about the procedure. Moreover, the patients walking speed (sometimes defined by researchers and sometimes by patient's choice) can interfere the meta-analysis if one study results are compared to another and probably does not reflect everyday walking characteristics of individuals [33–35]. Similarly, the application of footwear insoles with different sensor locations does not reflect the “daily work” of the foot because people walk at different speeds during everyday activities and use several types of footwear. The changes observed during standing are mainly the result of foot pathology, while during the walk, the abnormal image of pressure on the foot may be affected by other disorders: pain in the knees, hips, or spine [30]. Some authors [36] found that dynamic measurement is identical or even inferior to the static one because during walking, antalgic gait is promoted, so the real forces on the patients' soles could be misinterpreted. The static pedobarography could be helpful to detect structural changes within the foot without the influence of the other factors that may be revealed only during gait. The advantages and disadvantages of using different pressure measurement devices based on the current literature were clearly discussed and commented by Fernando and coauthors [30].

Although the value of both peak plantar pressure and pressure-time integral is reported in most of the studies, the systematic review by Bus and Waaijman showed that the added value of reporting pressure-time integral data is limited [37]. One of the most advanced peak plantar pressure classifications based on the results from pressure platform is the assessment done by Bennetts et al. [38]. The authors proposed regional pressure distribution for a total number of clusters set to seven, but as mentioned by the authors of this work, such mapping of the soles is possible only for specialists and difficult to use in everyday practice. Moreover, in this study, the obtained results were not compared to the physiological model, which may make difficult to interpret the result obtained in daily practice for the physician. In 2016, Deschamps et al. proposed the plantar pressure-based classification system in diabetic foot medicine [39]. This classification takes into consideration the possible 4 patterns for the highest pressure location, and the differences between the four clusters were coded with colour. The analysis was based on gait assessment, and within the examined patients were also subjects with previous (but not active) foot ulcer.

Static pedobarography, which is easier to administer for patients and medical staff than dynamic one, gives us important and sufficient data about foot structure and function [30, 40, 41]. In such cases, the pictures from static pedobarography could be helpful to detect abnormal distribution of plantar pressure which results from invisible foot structure deformity. This way, it can become a simple tool in the daily practice of diagnosing patients with diabetes [42].

The intensity of print colours, according to the device's software, without pressure value from static pedobarography analysis shows us abnormal load distribution on the soles which may predispose to calluses and those to ulcers, especially due to the loss of plantar pad thickness in these places [43]. The regions of high pressure are marked using “warm” colours (red or yellow), and the regions of low pressure are marked using “cold” colours (blue or green). This simply facilitates the identification of places of abnormal plantar loading distribution that can be corrected by insoles, injection of liquid silicone, or surgery interventions [44, 45]. The other pathologies within the foot are also more related to the distribution of the plantar pressure than to the absolute values of the pressure, e.g., forefoot pain [46]. Thus, it appears that the knowledge of the absolute pressure is not necessary to confirm the existing dysfunction within the foot structure.

In the last years, innovation like local temperature monitoring in the prevention of foot ulcer is proposed [47]. An unquestionable advantage of this technique is its objectivity and ease of performance. Nevertheless, the temperature rise seems to occur just before the development of the ulcer according to the five classical signs of inflammation (heat, pain—this can be masked by neuropathy, redness, swelling, and loss of function). Analysis of plantar pressures seems to indicate earlier disorders, when inflammation is not yet occurred.

The aim of our study was to evaluate the prevalence of different regions for the highest plantar pressure (HP) on the sole among patients with diabetes, based on images from static pedobarography interpreted every day in clinical practice and to explore its possible association with selected, available factors among patients with diabetes without previous diabetic foot diagnosis.

## 2. Material and Methods

**Figure 1:**
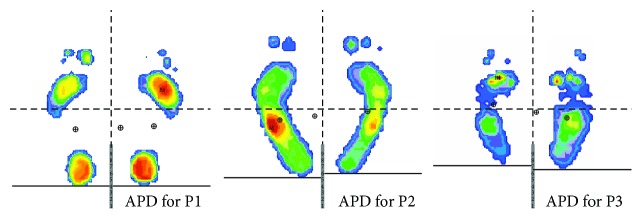
Examples of abnormal plantar pressure distribution (APD) for P1, P2, and P3.

We retrospectively investigated the distribution of the regions of the highest plantar pressure defined as a static, peak-plantar pressure. Then, we assessed the prevalence of the APD (abnormal pressure distribution) which was defined as the warmest colour obtained in the pedobarographic image, in a place on the sole other than physiological (defined according to tripod theory mentioned in Introduction). The results were obtained by colourful print analysis (Figure 1) among patients with diabetes mellitus (DM), and the association between this pressure and selected factors: neuropathy, gender, age, and BMI (body mass index) was also explored. Patients were qualified as APD positive (+) only if symmetrical APD was observed, as neuropathy, age, BMI, or gender, which were analysed in the context of their impact on APD, potentially have an impact on both feet.

### 2.1. Patients

Nine hundred seventy-four medical records of nonulcer patients with DM were retrospectively analysed. All of the documents which were obtained from the Diabetic Foot Centre (DFC), where the patients had consultations, covered a period of fifteen months of work.

Inclusion criteria: cases were defined as subjects who had a diagnosis of DM, live in the city where the DFC exists (consultations are sponsored from the city budget), and came spontaneously to be examined despite the absence of neuropathy signs or symptoms. None of the patients had previous foot ulcerations or operative procedures involving the foot. The evaluated group was representative of a large urban area in the country.

### 2.2. Examination and Data Subdivision

According to the standards of studies and papers on the prevention and management of foot ulcers in diabetes [31], we analysed necessary data dedicated to the lower extremity, coming from patients' medical records. A typical foot examination in the DFC consists of the ankle-brachial pressure index (ABPI), visible foot deformities, calluses, peripheral neuropathy, and plantar pressure assessment. The examination is always carried out at room temperature ranging from 24°C to 26°C. In addition to the foot examination, BMI, age, and gender data are collected.

Related to the physiological possible changes in foot structure [48] in the study, we subdivided patients into six groups to analyse the abnormal plantar pressure distribution (APD) with respect to age.

To study the prevalence of the APD among patients with different BMI, we subdivided subjects for two groups: patients with BMI ≥ 35 kg/m^2^ and with BMI < 35 kg/m^2^. We expected that severe obesity (BMI ≥ 35) can influence the plantar pressure pattern.

ABPI analysis was not taken into account in this part of the study as it is not connected with APD and, therefore, has been omitted in the following text.

Peripheral neuropathy: motor component of the peripheral neuropathy causes muscle atrophy within the feet with subsequent abnormal distribution of the plantar pressure, feet deformity, and calluses [49]. Information about visible deformities and calluses were derived from physical examination. Deformities included hammer or claw toes, hallux valgus, visible flat feet, or “other visible deformities”.

Calluses were defined as thick, hardened layers of the skin.

Peripheral neuropathy was assessed with questions (see below) and clinical evaluation in accordance with the local recommendations, and the tools used in the centre are similar to those recommended in the document prepared by Jeffcoate and coauthors [31]. A skilled, board-certified, nurse asked patients about stinging, numbness, tingling, or burning of the foot for the questionnaire items. Ten-gram monofilament and tuning fork (128 MHz) tests were administered. Monofilament was applied in 10 locations on the sole (calluses were avoided) and one on the dorsal part of the foot for checking the loss of protective sensation. A positive monofilament test was considered to be the lack of sensation of tightness in at least 6 of 11 tested sites. The tuning fork was applied for vibration detection to both ankles, the first metatarsophalangeal joint, and the anterior aspect of the shin bone sites. A positive vibration test was considered to be no detection of vibration in three of four test sites [50].

Two positive test results and typical symptoms of neuropathy were the basis for confirmation of symmetric, peripheral, sensory polyneuropathy based on the local, internal guidelines in the centre. The condition required for the occurrence of these disorders was symmetry. Analyzing the current knowledge of the principles of diagnosis of sensory diabetic neuropathy as a risk factor for foot ulcers, these tests are sufficient for its identification. International Working Group on the Diabetic Foot [51] recommend monofilament, tuning fork, and cotton wool tests for sensory neuropathy detection, while data from PODUS [52] and Inlow's 60-second Diabetic Foot Screen [53, 54] suggest only monofilament test, as representative for damage to the sensory components (in combination with interview) to assess risk for foot ulceration.

All of the above information that was analysed came from history cards held by skilled diabetes nurse with several years of experience in the study of feet in patients with diabetes and subsequently confirmed by a physician, if in doubt.

Pedobarograms: the PEL-38 Medicapteurs SAS device, Balma, France (https://www.medicapteurs.com/diabetic-tool/), is used in DFC. The patient stands on a special platform with pressure sensors connected to the computer to produce a static pressure profile. If necessary, the patient repeats the test until a correct impression of the foot (symmetric with the corresponding location of the centre of the mass) to avoid, consciously or subconsciously, off-loading one foot, e.g., due to hip pain. This is a standard procedure in the DFC.

For the purpose of this analysis, the authors assessed plantar pressures using a semiquantitative method, like static barefoot pedobarographic records with colourful print analysis. The intensity of colour was proportional to the pressure received. Warm colours indicated the greatest pressure, while cold colours indicated the least plantar pressure (starting with red, then yellow, green, and blue). Pedobarograms from the centre were assessed, for the internal purpose of this study, by an independent physician (diabetologist), who was blind to the subjects' status. This physician has been previously trained in the evaluation of pedobarograms to the extent necessary for the study. Moreover, 20 randomly selected prints were similarly tested for verification by an orthopaedist with experience in this field (internal validation). The results were consistent at 100%. APDs were analysed for the forefoot (P1—about 25% of the foot length) and midfoot (P3—about 28% of the foot length), with a separate evaluation for the lateral (P2) part of the midfoot (the edge of the foot) (Figure 1). The length of the foot come from pedobarograms' documents and was measured as a line length with a ruler. This line connected two points: one end of the foot (the most forward point of the foot) to the other end of the foot—located on the heel. The line was run parallel to the central, vertical line visible on the mat (similar as is visible on the pedobarograms' pictures, Figure 1), and then its length was the basis to calculate the % of the foot length [55].

The load of the hallux (about 20% of foot length), which is a part of forefoot and rearfoot (about 27% of the foot length), was not analysed. In the assessment, we used colour intensity, not values of the pressure evaluation (semiquantitative method), to demonstrate the presence of the maximum pressure (peak pressure) represented by the hottest colour—e.g., the red one. For the heel, the hottest colour does not constitute pathology, because this colour always indicates a site of greatest pressure (typically presented within the heel according to the tripod theory). The heel load evaluation is therefore only useful if it includes absolute pressure value assessment.

### 2.3. Statistical Analysis

The Statistica 9 PL (StatSoft) software package was used for statistical analysis. The Kołmogorowa-Smirnowa test was used in the distribution analysis, according to the result of the analysis, the parametric, *T*-test, or nonparametric; *U* Mann-Whitney test was used in further calculations. The chi-square test was used to determine the association between two categorical variables. Data are presented as means (±S.D.). A *P* value < 0.05 was considered statistically significant.

The study was approved by the Commission of Bioethics at local Medical University.

## 3. Results

### 3.1. Population of the Study

The authors retrospectively analysed 974 medical records (974 history cards and 1948 feet pedobarograms from 451 males and 523 females). The mean patient age was 64.6 years (±11.1): 63.8 (±10.9) for men and 65.3 (±11.3) for women, *P* > 0.05. The mean BMI was 29.9 kg/m^2^ (±5.2): 29.4 (±4.7) for men and 30.4 (±5.6) for women, *P* > 0.05.

### 3.2. The Prevalence and the most Frequent Location of the APD according to the HP Analysis

In the cohort, 80 patients (8.21%) had a typical region of HP according to the tripod theory, with no APD (37 females and 43 males (7.07% and 9.53%, respectively)). In 894 cases (91.79%), at least one, symmetrical location of the APD was noted (P1 (*N* = 806) and/or P2 (*N* = 216) and/or P3 (*N* = 26))—most of the APDs were found within the forefoot (metatarsophalangeal joints); the least frequent location was within the midfoot.

### 3.3. Peripheral Neuropathy and APD

Peripheral, symmetric, sensory foot neuropathies (PSSN) were shown in 6.88% (*N* = 67) of the subjects. No association existed between the APD and the presence of the PSSN (Table 1).

The calluses and feet deformities were demonstrated in 33.78% (*N* = 329) and 18.07% (*N* = 176), respectively. Deformities and secondary calluses are considered an expression of motor neuropathy, which results in muscle weakness. The next stage of our study was to assess the relationship between these abnormalities and the presence of APD revealed during the pedobarographic examination. No association was found between the APD and the presence of calluses (*P* = 0.32), as well as between the APD and the presence of visible deformities (*P* = 0.17) (Table 1). In exceptional cases, the presence of callus or deformation was found without APD (*N* = 23 and *N* = 10, respectively), but many patients with APD had no calluses (*N* = 588) or visible deformities (*N* = 728).

### 3.4. APD and BMI

When authors assessed this relationship for BMI and APD without dividing for P1, P2, and P3, the *P* value was 0.27. Only after dividing the patients into groups, we noted that APD for P1 was more common in patients with a BMI < 35 kg/m^2^, while P2 and P3 were more common in the BMI ≥ 35 kg/m^2^ group (*P* = 0.0015, *P* < 0.0001, and *P* < 0.0001, respectively (Table 2)).

### 3.5. APD and Gender

Tripod load distribution within both feet existed with near-equal prevalence in both genders (43 males and 37 females (9.53% and 7.07%, respectively); *P* = 0.16). APD within the forefoot (P1) had a similar prevalence for males and females (84.26% and 81.45%, respectively; *P* = 0.25). APD on the lateral part of the foot (P2) and midfoot (P3) occurred significantly more often in females (*P* = 0.00066 and *P* = 0.005, respectively) (Table 3).

### 3.6. APD and Age

There was no association between age and the presence of the APD for total points (*P* = 0.44) (Table 4), as well as for P1, P2, and P3 separately (*P* = 0.4, *P* = 0.06, and *P* = 0.34, respectively).

## 4. Discussion

The consequence of pathological changes in the feet is a disturbance of standing and walking mechanics. The first one is evaluated during a static pedobarographic examination. For quick analysis of the foot structure, pedobarographic images are used in everyday medical practice [56]. Based on them, a decision is made on whether or not there is a need for specialist consultations (podologist, orthopedist) and/or insoles.

In our observational, descriptive, retrospective analysis, the pattern of loading across the sole showed that the most common region for the high plantar pressure was the central metatarsal heads (II-IV metatarsophalangeal joints). This area appears to be strongly associated with the formation of ulcers, e.g., Eurodiale study showed that about 55% of diabetic ulcers are located on patients' toes but 22% of all ulcers concerns the forefoot/midfoot area [57]. In other studies, APD and higher pressure (detected for feet calluses and deformities) led to ulcerations also, particularly at the height of the metatarsophalangeal joints [58–60] and even half of the plantar foot ulcers were described as located under metatarsal heads and hallux [57, 61, 62]. From a clinical point of view regardless of the foot inspection results, APD can be detected as the first pathology which precedes visible abnormality.

The most common type of neuropathy in the population of patients with diabetes is peripheral, sensorimotor, symmetric polyneuropathy [63]. The sensory and the motor neuropathy both play an important role in the foot ulcer formation [22, 26, 64–67]. According to our very strict criteria, the sensory component of this disorder occurred in nearly 7% of the subjects, but visible deformation (18.07%) and calluses (33.78%) (resulting from motor component of the neuropathy) were more frequent. The above findings may result from the fact that the motor disturbances can be more common than sensory as was shown by Ishpekova et al. [68] or that foot deformation and/or calluses can occur independently of the peripheral neuropathy. According to Farndon [69], there was no statistically significant difference in diabetic versus nondiabetic patients concerning the incidence of toe deformity (claw/hammer toes), although the prevalence of sensory neuropathy was significantly greater in the diabetic population. Data from the other study also showed that neuropathy is not simply related to calluses, foot deformities, or joint mobility [70]; however, this neurologic, motor-related pathologies are dangerous for patients with diabetes as can provoke injury.

To summarise the connections between neuropathy and abnormal pressure distribution, the major finding in our study of patients with DM was that sensory and equivalents of motor components of the peripheral neuropathy were not connected with APD (APD was more common than PSSN, calluses, and foot deformity) which was also mentioned in the literature [71]. Considering the natural course of the disease, it could be that abnormal pressure distribution can be found before peripheral neuropathy detected by routine tests. Dinh and Veves [61] in their review also summarised that increase of peak pressures within the forefoot could be the first observations in the absence of any detectable signs or symptoms of neuropathy which is the consequence of the progressive nature of the disease.

We also connected the different loading points with gender, BMI, and age. Generally, as in the previous study [72], the authors of this one also did not find an association of gender to the plantar pressure distribution. The only difference was that while the pathologies within the forefoot were found in a similar number of males and females, the APD for the lateral part of the foot and midfoot was found more often in females. Hills et al. [72] also demonstrated an increase in pressure under the midfoot for obese females as compared to obese males. For females, a slightly higher BMI was demonstrated in our study so the authors can only speculate that an increased body mass generates overload in these locations.

The result for the relationship between APD for forefoot and BMI was unexpected. The more frequent occurrence of APD in this location in people with a lower BMI indicates the participation of factors other than weight in the formation of forefoot overload. For midfoot and the lateral part of the foot, the APD was found more frequently for BMI equal or higher than 35 kg/m^2^, as we expected. Despite the connection between the BMI and the value of the plantar pressure [3], our findings suggest that in APD, forefoot identification, BMI as the most important factor, should not be considered. People with BMI lower than 1st degree (according to the WHO description) of the obesity seem to be at the higher risk for forefoot overload.

This work was not intended to propose a new classification of pressure distribution on the sole (pressure mapping) but to assess the prevalence of the various regions of the highest peak plantar pressure with indication of the abnormal location of this pressure beneath the plantar surface (without defining its value) represented by colour mapping, in everyday practice. Such visual assessment is simple and understandable for both the primary care physician and the patient [56], which facilitates its use in everyday practice. The impact of pressure pattern on foot ulcer location will be mandatory in the future to determine whether there are correlations between this two.

In the study, we do not refer to healthy population, because such a population was not examined in the DFC. For this reason, to define the potentially incorrect pressure location on the sole, we referred to tripod theory based on Taha et al.'s [2] observation.

The lack of our study is that the evaluation of the neuropathy in the Diabetic Foot Centre was based on local recommendation and not on, e.g., Michigan Neuropathy Screening Instrument (MNSI). However, as a research tool in the mentioned centre, foot inspection, vibration sensation, monofilament testing, and questionnaire for symptoms were used. These tools were similar to the MNSI as well as mentioned in reporting standards prepared by Jeffcoate and coauthors [31].

Because of the retrospective nature of the study, unfortunately, it was not possible to carry out this analysis for the different types and duration of DM due to the lack of complete data in the history of the disease coming from the DFC. This is why for such a big number of patients, we took into consideration only the parameters of interest which were available for all consulted patients within the mentioned period. The baseline characteristics of the study population appear to be typical for subjects with type 2 diabetes (due to BMI and age), and the results could change for different types of DM. The disease duration may be connected with higher peak pressure within the feet due to the plantar contact area narrowing (shown in dynamic evaluation) [73]. Mayfield et al. [74] found that age and duration of diabetes are connected with ulcers and amputations so it cannot be ruled out that both could also influence the pressure distribution. However, we should recognize that in type 2 DM, the known duration of the disease is only approximate, so diabetes duration is a quite frequent problem which biases study results. In the study, we demonstrate that the age of patients with DM does not affect the presence of APD. This indicates a need to consider APD testing regardless of patient age.

The limitations of our study, in addition to the aforementioned, are mainly related to a semiquantitative analysis of the pressure map, the retrospective nature of the study, and the nature of the centre. Although the authors involved patients from only one centre of a large urban area, the available data seem to be representative of the entire diabetic population. The strength of this study is its large size and uniform assessment of the neuropathy even if not strictly relating to MNSI. As we analysed the information from barefoot print, it also should be taken into consideration that this analysis does not provide us with information about the interaction between foot and footwear. It is mean that potentially “healthy” people may also have problems if they wear unsuitable shoes.

Relieving pressure points and avoiding callus formation are still the basic goal in patient care. The ability to visualize the focal pressures under the foot as easy-to-red, colour-coded diagrams can facilitate patient training and education [56]. This simple low-cost static pressure analysis also provides the clinician with information about a possible intervention, e.g., the surgical or application of the insoles [75]. Although there is no clear evidence that off-loading is important in the prevention of primary foot ulcers in diabetic patients, as highlighted in the Cavanagh and Bus review [76], in everyday practice, it is unethical to avoid actions that are aimed at improving the pressure distribution on the sole. In the absence of a clinical gold standard, the current approach in the choice of simple pressure map analysis remains an important part of the patient care. Because the lack of standard practices on this field may limit clinical use, so further validity and reliability of the colour intensity measure of plantar pressure is required.

## 5. Conclusions

The prevalence of the abnormal plantar pressure distribution when applied, the tripod theory was high in this analysis. The most common location of the highest plantar pressure was the central part of the forefoot. Female gender and BMI ≥ 35 predispose to the lateral part and midfoot abnormal pressure distribution, whereas other, unsearchable factors are responsible for APD of the forefoot. Connections between calluses or deformation after months/years of duration of asymptomatic APD need to be identified.

Because the prevalence of the abnormal plantar pressure distribution among patients with diabetes is high and dynamic measurements are much more time consuming and expensive, the simple colourful print analysis should be recognized as a helpful tool in identifying invisible pathology on the sole in each patient. There is no simple relationship between the clinical-available variables and APD so such analysis can help practitioners to choose the appropriate prophylaxis.

## Figures and Tables

**Table 1:** Association between APD and PSSN on physical examination; the presence of calluses and visible deformities.

NT (%)	Patients with abnormal planter pressure distribution (%)	Patients without abnormal planter pressure distribution (%)	NT	*P*
Patients without peripheral symmetric sensory neuropathy	833 (91.8)	74 (8.2)	907	0.82
Patients with peripheral symmetric sensory neuropathy	61 (91.0)	6 (9.0)	67	
Patients without calluses	588 (91.2)	57 (8.8)	645	0.32
Patients with calluses	306 (93.0)	23 (7.0)	329	
Patients without foot deformity	728 (91.2)	70 (8.8)	798	0.17
Patients with foot deformity	166 (94.3)	10 (5.7)	176	

A *P* value of < 0.05 is considered statistically significant. Data are presented as number (percentage). NT: total number of patients, APD: abnormal plantar pressure distribution, PSSN: peripheral, symmetric, sensory foot neuropathy.

**Table 2:** Association between APD for the forefoot, lateral part of the foot, midfoot, and BMI.

NT (%)	Patients without abnormal plantar pressure distribution within the forefoot (%)	Patients with abnormal plantar pressure distribution within the forefoot (%)	Patients without abnormal plantar pressure distribution within the lateral part of the foot (%)	Patients with abnormal plantar pressure distribution within the lateral part of the foot (%)	Patients without abnormal plantar pressure distribution within the midfoot (%)	Patients with abnormal plantar pressure distribution within the midfoot (%)
BMI < 35	128 (15.6)	693 (84.4)	665 (81.0)	156 (19.0)	808 (98.4)	13 (1.6)
BMI ≥ 35	40 (26.1)	113 (73.9)	93 (60.8)	60 (39.2)	140 (91.5)	13 (8.5)
*P*	*0.0015*		*<0.0001*		*<0.0001*	

A *P* value of <0.05 is considered statistically significant. Data are presented as number (percentage). BMI: body mass index; NT: total number of patients; APD: abnormal plantar pressure distribution.

**Table 3:** Prevalence of APD in the forefoot, lateral part of the foot, and midfoot for gender.

NT	Abnormal plantar pressure distribution within the forefoot (%)	Abnormal plantar pressure distribution within the lateral part of the foot (%)	Abnormal plantar pressure distribution within the midfoot (%)
Male *n* = 451	380 (84.3)	78 (17.3)	5 (1.1)
Female *n* = 523	426 (81.4)	138 (26.4)	21 (4.0)
NT = 974	806	216	26
*P*	0.25	*0.00066*	*0.005*

A *P* value of <0.005 is considered statistically significant. Data are presented as number (percentage). NT: total number of patients. APD: abnormal plantar pressure distribution.

**Table 4:** Prevalence of APD for age.

Age (years)	NT	Patients without abnormal plantar pressure distribution (%)	Patients with abnormal plantar pressure distribution (%)
→40	36	5 (13.9)	31 (86.1)
41-50	51	3 (5.9)	48 (94.1)
51-60	233	15 (6.4)	218 (93.6)
61-70	333	28 (8.4)	305 (91.6)
71-80	269	22 (8.2)	247 (91.8)
81-90	52	7 (13.6)	45 (86.5)
NT	974	80	894
*P*	0.44		

A *P* value of <0.005 is considered statistically significant. Data are presented as number (percentage). NT: total number of patients. APD: abnormal plantar pressure distribution.

## Data Availability

The data used to support the findings of this study are available from the corresponding author upon request.

## Additional Points

*Key Messages*. (1) The prevalence of the abnormal regions for the highest pressure on the sole, among patients with diabetes, is very high. (2) The simple colourful print analysis should be recognised as a helpful tool in identifying invisible pathology on the sole in each patient. (3) The standard physical examination (inspection) and tests (vibration, monofilament tests) could be not sufficient in everyday practice to avoid foot ulceration.

## References

[1] Cavanagh P. R., Rodgers M. M., liboshi A. (1987). Pressure distribution under symptom-free feet during barefoot standing. *Foot & Ankle*.

[2] Taha Z., Norman M. S., Omar S. F. S., Suwarganda E. (2016). A finite element analysis of a human foot model to simulate neutral standing on ground. *Procedia Engineering*.

[3] Hotfiel T., Carl H. D., Wendler F., Jendrissek A., Heiß R., Swoboda B. (2017). Plantar pressures increase with raising body weight: a standardised approach with paired sample using neutral shoes. *Journal of Back and Musculoskeletal Rehabilitation*.

[4] Martinez-Nova A., Huerta J. P., Sánchez-Rodriguez R. (2008). Cadence, age, and weight as determinants of forefoot plantar pressures using the biofoot in-shoe system. *Journal of the American Podiatric Medical Association*.

[5] Rao S., Saltzman C., Yack H. J. (2006). Ankle ROM and stiffness measured at rest and during gait in individuals with and without diabetic sensory neuropathy. *Gait & Posture*.

[6] Orendurff M. S., Rohr E. S., Sangeorzan B. J., Weaver K., Czerniecki J. M. (2006). An equinus deformity of the ankle accounts for only a small amount of the increased forefoot plantar pressure in patients with diabetes. *The Journal of Bone & Joint Surgery*.

[7] Couppé C., Svensson R. B., Kongsgaard M. (2016). Human Achilles tendon glycation and function in diabetes. *Journal of Applied Physiology*.

[8] Barn R., Waaijman R., Nollet F., Woodburn J., Bus S. A. (2015). Predictors of barefoot plantar pressure during walking in patients with diabetes, peripheral neuropathy and a history of ulceration. *PLoS One*.

[9] Boulton A. J. M., Hardisty C. A., Betts R. P. (1983). Dynamic foot pressure and other studies as diagnostic and management aids in diabetic neuropathy. *Diabetes Care*.

[10] Boulton A. J. M., Betts R. P., Franks C. I., Newrick P. G., Ward J. D., Duckworth T. (1987). Abnormalities of foot pressure in early diabetic neuropathy. *Diabetic Medicine*.

[11] Duckworth T., Boulton A. J., Betts R. P., Franks C. I., Ward J. D. (1985). Plantar pressure measurements and the prevention of ulceration in the diabetic foot. *The Journal of Bone & Joint Surgery*.

[12] Stokes I. A. F., Faris I. B., Hutton W. C. (1975). The neuropathic ulcer and loads on the foot in diabetic patients. *Acta Orthopaedica Scandinavica*.

[13] Bacarin T. A., Sacco I. C. N., Hennig E. M. (2009). Plantar pressure distribution patterns during gait in diabetic neuropathy patients with a history of foot ulcers. *Clinics*.

[14] Boulton A. J. (1987). The importance of abnormal foot pressures in early diabetic neuropathy. *Diabetic Medicine*.

[15] Rich J., Veves A. (2000). Foorfoot and rearfoot plantar pressures in diabetic patients: correlation to foot ulceration. *Wounds*.

[16] Cavanagh P. R., Morag E., Boulton A. J. M., Young M. J., Deffner K. T., Pammer S. E. (1997). The relationship of static foot structure to dynamic foot function. *Journal of Biomechanics*.

[17] Lavery L. A., Armstrong D. G., Wunderlich R. P., Tredwell J., Boulton A. J. M. (2003). Predictive value of foot pressure assessment as part of a population-based diabetes disease management program. *Diabetes Care*.

[18] Bus S. A., Maas M., de Lange A., Michels R. P. J., Levi M. (2005). Elevated plantar pressures in neuropathic diabetic patients with claw/hammer toe deformity. *Journal of Biomechanics*.

[19] Pham H., Armstrong D. G., Harvey C., Harkless L. B., Giurini J. M., Veves A. (2000). Screening techniques to identify people at high risk for diabetic foot ulceration: a prospective multicenter trial. *Diabetes Care*.

[20] Boulton A. J., Betts R. P., Franks C. I., Ward J. D., Duckworth T. (1987). The natural history of foot pressure abnormalities in neuropathic diabetic subjects. *Diabetes Res*.

[21] Frykberg R. G., Lavery L. A., Pham H., Harvey C., Harkless L., Veves A. (1998). Role of neuropathy and high foot pressures in diabetic foot ulceration. *Diabetes Care*.

[22] Fernando M. E., Crowther R. G., Pappas E. (2014). Plantar pressure in diabetic peripheral neuropathy patients with active foot ulceration, previous ulceration and no history of ulceration: a meta-analysis of observational studies. *PLoS One*.

[23] Masson E. A., Hay E. M., Stockley I., Veves A., Betts R. P., Boulton A. J. M. (1989). Abnormal foot pressures alone may not cause ulceration. *Diabetic Medicine*.

[24] van Schie C. H. M. (2005). A review of the biomechanics of the diabetic foot. *The International Journal of Lower Extremity Wounds*.

[25] van Schie C. H. M., Boulton A. J. M., Veves A., Giurini J. M., LoGerfo F. W. (2002). Biomechanics of the diabetic foot: the road to foot ulceration. *The Diabetic Foot*.

[26] Veves A., Murray H. J., Young M. J., Boulton A. J. M. (1992). The risk of foot ulceration in diabetic patients with high foot pressure: a prospective study. *Diabetologia*.

[27] Wrobel J. S., Najafi B. (2010). Diabetic foot biomechanics and gait dysfunction. *Journal of Diabetes Science and Technology*.

[28] Weijers R. E., Walenkamp G. H. I. M., van Mameren H., Kessels A. G. H. (2003). The relationship of the position of the metatarsal heads and peak plantar pressure. *Foot & Ankle International*.

[29] Duckworth T., Betts R. P., Franks C. I., Burke J. (1982). The measurement of pressures under the foot. *Foot and Ankle International*.

[30] Fernando M. E., Crowther R. G., Wearing S., Müller B., Wolf S. I. (2018). The importance of foot pressure in diabetes. *Handbook of Human Motion*.

[31] Jeffcoate W. J., Bus S. A., Game F. L., Hinchliffe R. J., Price P. E., Schaper N. C. (2016). Reporting standards of studies and papers on the prevention and management of foot ulcers in diabetes: required details and markers of good quality. *The Lancet Diabetes & Endocrinology*.

[32] Arts M. L. J., Bus S. A. (2011). Twelve steps per foot are recommended for valid and reliable in-shoe plantar pressure data in neuropathic diabetic patients wearing custom made footwear. *Clinical Biomechanics*.

[33] Segal A., Rohr E., Orendurff M., Shofer J., O'Brien M., Sangeorzan B. (2004). The effect of walking speed on peak plantar pressure. *Foot & Ankle International*.

[34] Burnfield J. M., Few C. D., Mohamed O. S., Perry J. (2004). The influence of walking speed and footwear on plantar pressures in older adults. *Clinical biomechanics*.

[35] Chung M. J., Wang M. J. (2012). Gender and walking speed effects on plantar pressure distribution for adults aged 20–60 years. *Ergonomics*.

[36] Choi Y. R., Lee H. S., Kim D. E., Lee D. H., Kim J. M., Ahn J. Y. (2014). The diagnostic value of pedobarography. *Orthopedics*.

[37] Bus S. A., Waaijman R. (2013). The value of reporting pressure-time integral data in addition to peak pressure data in studies on the diabetic foot: a systematic review. *Clinical Biomechanics*.

[38] Bennetts C. J., Owings T. M., Erdemir A., Botek G., Cavanagh P. R. (2013). Clustering and classification of regional peak plantar pressures of diabetic feet. *Journal of Biomechanics*.

[39] Deschamps K., Matricali G. A., Desmet D. (2016). Efficacy measures associated to a plantar pressure based classification system in diabetic foot medicine. *Gait & Posture*.

[40] Cavanagh P. R., Rodgers M. M. (1987). The arch index: a useful measure from footprints. *Journal of Biomechanics*.

[41] Zulkifli S. S., Loh W. P. (2018). A state-of-the-art review of foot pressure. *Foot and Ankle Surgery*.

[42] Lalande X., Vie B., Weber J. P., Jammes Y. (2016). Normal values of pressures and foot areas measured in the static condition. *Journal of the American Podiatric Medical Association*.

[43] Dalal S., Widgerow A. D., Evans G. R. D. (2015). The plantar fat pad and the diabetic foot - a review. *International Wound Journal*.

[44] van Schie C. H., Whalley A., Vileikyte L., Wignall T., Hollis S., Boulton A. J. (2000). Efficacy of injected liquid silicone in the diabetic foot to reduce risk factors for ulceration: a randomized double-blind placebo-controlled trial. *Diabetes Care*.

[45] van Schie C. H., Whalley A., Vileikyte L., Boulton A. J. (2002). Efficacy of injected liquid silicone is related to peak plantar pressures in the neuropathic diabetic foot. *Wounds*.

[46] Keijsers N. L. W., Stolwijk N. M., Louwerens J. W. K., Duysens J. (2013). Classification of forefoot pain based on plantar pressure measurements. *Clinical Biomechanics*.

[47] Bus S. A. (2016). Innovations in plantar pressure and foot temperature measurements in diabetes. *Diabetes/Metabolism Research and Reviews*.

[48] Scott G., Menz H. B., Newcombe L. (2007). Age-related differences in foot structure and function. *Gait & Posture*.

[49] Zimny S., Schatz H., Pfohl M. (2004). The role of limited joint mobility in diabetic patients with an at-risk foot. *Diabetes Care*.

[50] Perkins B. A., Olaleye D., Zinman B., Bril V. (2001). Simple screening tests for peripheral neuropathy in the diabetes clinic. *Diabetes Care*.

[51] Bakker K., Apelqvist J., Lipsky B. A., van Netten J. J., Schaper N. C., on behalf of the International Working Group on the Diabetic Foot (IWGDF) (2016). The 2015 IWGDF guidance documents on prevention and management of foot problems in diabetes: development of an evidence-based global consensus. *Diabetes/Metabolism Research and Reviews*.

[52] Crawford F., Cezard G., Chappell F. M. (2015). A systematic review and individual patient data meta-analysis of prognostic factors for foot ulceration in people with diabetes: the international research collaboration for the prediction of diabetic foot ulcerations (PODUS). *Health Technology Assessment*.

[53] Inlow S. (2004). The 60-second foot exam for people with diabetes. *Wound Care Canada*.

[54] Carreau L., Niezgoda H., LeBlond S., Trainor A., Orsted H., Woodbury M. G. (2013). A prospective, descriptive study to assess the reliability and usability of a rapid foot screen for patients with diabetes mellitus in a complex continuing care setting. *Ostomy/Wound Management*.

[55] Gurney J. K., Kersting U. G., Rosenbaum D. (2008). Between-day reliability of repeated plantar pressure distribution measurements in a normal population. *Gait & Posture*.

[56] Gurney J. K., Kersting U. G., Rosenbaum D. (2017). Pedobarography as a clinical tool in the management of diabetic feet in New Zealand: a feasibility study. *Journal of Foot and Ankle Research*.

[57] Prompers L., Huijberts M., Apelqvist J. (2007). High prevalence of ischaemia, infection and serious comorbidity in patients with diabetic foot disease in Europe. Baseline results from the Eurodiale study. *Diabetologia*.

[58] Bowling F. L., Reeves N. D., Boulton A. J. (2011). Gait-related strategies for the prevention of plantar ulcer development in the high risk foot. *Current Diabetes Reviews*.

[59] Perell K. L., Merrill V., Nouvong A. (2006). Location of plantar ulcerations in diabetic patients referred to a Department of Veterans Affairs podiatry clinic. *Journal of Rehabilitation Research and Development*.

[60] Ledoux W. R., Shofer J. B., Cowley M. S., Ahroni J. H., Cohen V., Boyko E. J. (2013). Diabetic foot ulcer incidence in relation to plantar pressure magnitude and measurement location. *Journal of Diabetes and its Complications*.

[61] Dinh T. L., Veves A. (2005). A review of the mechanisms implicated in the pathogenesis of the diabetic foot. *The International Journal of Lower Extremity Wounds*.

[62] York R. M., Perell-Gerson K. L., Barr M., Durham J., Roper J. M. (2009). Motor learning of a gait pattern to reduce forefoot plantar pressures in individuals with diabetic peripheral neuropathy. *PM&R*.

[63] Bakker K., Schaper N. C., on behalf of the International Working Group on the Diabetic Foot Editorial Board (2012). The development of global consensus guidelines on the management and prevention of the diabetic foot 2011. *Diabetes/Metabolism Research and Reviews*.

[64] Armstrong D. G. (2005). Detection of diabetic peripheral neuropathy: strategies for screening and diagnosis. *Advanced Studies in Medicine*.

[65] Wood W. A., Wood M. A., Werter S. A. (2005). Testing for loss of protective sensation in patients with foot ulceration: a cross-sectional study. *Journal of the American Podiatric Medical Association*.

[66] Mueller M. J., Minor S. D., Sahrmann S. A., Schaaf J. A., Strube M. J. (1994). Differences in the gait characteristics of patients with diabetes and peripheral neuropathy compared with age-matched controls. *Physical Therapy*.

[67] Fernando M. E., Crowther R. G., Lazzarini P. A., Sangla K. S., Buttner P., Golledge J. (2016). Gait parameters of people with diabetes-related neuropathic plantar foot ulcers. *Clinical Biomechanics*.

[68] Ishpekova B., Daslov M., Muradyan N., Alexandrov A. (2007). Clinical and electrophysiologi-cal studies in diabetic polyneuropathy. *Acta Medica Bulgarica*.

[69] Farndon L. J. (2000). The incidence of claw toes in diabetic and non-diabetic patients in a podiatry department. *Practical Diabetes International*.

[70] Lázaro-Martínez J. L., Aragón-Sánchez F. J., Beneit-Montesinos J. V., González-Jurado M. A., Morales E. G., Hernández D. M. (2011). Foot biomechanics in patients with diabetes mellitus: doubts regarding the relationship between neuropathy, foot motion, and deformities. *Journal of the American Podiatric Medical Association*.

[71] Skopljak A., Muftic M., Sukalo A., Masic I., Zunic L. (2014). Pedobarography in diagnosis and clinical application. *Acta Informatica Medica*.

[72] Hills A. P., Hennig E. M., McDonald M., Bar-Or O. (2001). Plantar pressure differences between obese and non-obese adults: a biomechanical analysis. *International Journal of Obesity and Related Metabolic Disorders*.

[73] Tuna H., Birtane M., Guldiken S. (2014). The effect of disease duration on foot plantar pressure values in patients with type 2 diabetes mellitus. *Türkiye Fiziksel Tip ve Rehabilitasyon Dergisi*.

[74] Mayfield J. A., Reiber G. E., Sanders L. J., Janisse D., Pogach L. M. (1998). Preventive foot care in people with diabetes. *Diabetes Care*.

[75] Bus S. A., van Deursen R. W., Armstrong D. G. (2016). Footwear and offloading interventions to prevent and heal foot ulcers and reduce plantar pressure in patients with diabetes: a systematic review. *Diabetes/Metabolism Research and Reviews*.

[76] Cavanagh P. R., Bus S. A. (2010). Off-loading the diabetic foot for ulcer prevention and healing. *Journal of Vascular Surgery*.

